# Relationship Between Lipodystrophy, Body Composition, Metabolic Profile, and Serum Levels of Adipocytokines

**DOI:** 10.3389/fnut.2021.750721

**Published:** 2021-12-09

**Authors:** Lívia Bertazzo Sacilotto, Silvia Justina Papini, Adriana Lucia Mendes, Mariana Gatto, Paulo Câmara Marques Pereira, José Eduardo Corrente, Julhiany de Fátima da Silva

**Affiliations:** ^1^Department of Infectology, Dermatology, Diagnostic Imaging, and Radiotherapy, Botucatu Medical School, São Paulo State University (UNESP), São Paulo, Brazil; ^2^Department of Nursing, Botucatu Medical School, São Paulo State University (UNESP), São Paulo, Brazil; ^3^Internal Medicine Department, Botucatu Medical School, São Paulo State University (UNESP), São Paulo, Brazil; ^4^Department of Biostatistics, Institute of Biosciences, São Paulo State University (UNESP), São Paulo, Brazil

**Keywords:** adipocytes, body composition, HIV, lipodystrophy, metabolic syndrome

## Abstract

**Background:** Despite the benefits in improving the clinical state of people living with HIV/aids (PLWHA), some side effects associated with the use of antiretroviral therapy (ART) are reported. Redistribution of body fat has been associated with treatment and is characterized by morphological changes, also known as lipodystrophy. The complications of metabolic and morphological changes in these individuals seem to increase the risk of cardiovascular disease. Adipocytokines are proteins that have essential functions in biological processes, in which the levels of these proteins are related to the pathogenesis of metabolic syndrome (MS) and cardiovascular disease. Recent studies have shown that such levels are generally modified in PLWHA, regardless of whether the treatment is established or not. An application of methods for body fat estimation in patients with fat redistribution, as in the case of aids, especially those that quantify body fat by segments, appears to clarify these alterations and plays an important role in the development of multiprofessional treatment.

**Objectives:** This investigation was carried out to compare and correlate body composition, biochemical metabolic parameters, and levels of adipocytokines and cytokines of PLWHA, with and without lipodystrophy, with individuals with negative HIV serology and stratified by sex.

**Material and Methods:** This is a cross-sectional study in which body composition, metabolic and anthropometric changes, and levels of adipocytokines of 110 individuals were assessed. These individuals were paired in sex, age, and body mass index (BMI) and subdivided into three groups: PLWHA with and without a clinical diagnosis of lipodystrophy associated with HIV, and a group control.

**Results:** Collinearity was identified both in the general sample and for genders of the waist-to-height ratio (WHtR) with all anthropometric parameters, except for muscle mass. The results show strong association between IFN-γ and TNF-α both in the general sample and for genders and moderate correlation between leptin and fasting glucose for women; worsening of the triglyceride profile in both women with lipodystrophy compared with the control group and men without lipodystrophy compared with the control group; higher serum TNF-α values among men without lipodystrophy compared to those with HIV-associated lipodystrophy (HALS).

**Conclusions:** The results of this study underline that, considering the manifestations of the syndrome, these patients have a high-risk endocrine metabolic profile for cardiovascular events.

## Introduction

The increase in life expectancy and life quality improvement of people living with HIV/AIDS (PLWHA) is directly due to the development of antiretroviral drugs and access to specific combined therapies, as they contribute to a significant reduction in morbidity and mortality associated with acquired immunodeficiency syndrome (AIDS) (1, 2). Since 1996, Brazil has provided universal access to free antiretroviral therapy (ART), and as a consequence, survival rate of patients infected with HIV/AIDS has improved dramatically. Since 2017, the country's national preferred first-line regimen has been dolutegravir (DTG) + lamivudine (3TC) + tenofovir (TDF). The preferred second-line regimen is TDF + 3TC + efavirenz (EFV) (as of 2014, as a three-in-one pill simplifying ART and enhancing adherence) (3). The evolution of the drugs available is high related to adherence rate and collateral effects, once the toxicity has been moderate belonging to the years.

Since specific treatment for the disease is available and there was a reduction in mortality rates, it was possible to observe that AIDS has become a treatable chronic disease. Similarly, there has been advance in the development of treatment over time as several studies have identified important adverse events associated with the continuous use of ART, such as the HIV-associated lipodystrophy (HALS) syndrome (4–7).

The HALS syndrome is characterized by a set of morphological changes in the distribution of body fat and/or metabolic abnormalities, including lipodystrophy, dyslipidemia, insulin resistance, and hyperglycemia (8–11). HALS is characterized by a redistribution of body fat in PLWHA and can be classified according to the region where accumulation and/or reduction in body fat occurs. Although the pathogenesis of HALS is not yet fully understood, it is known to be multifactorial and probably the result of the interaction between ART, viral infection, genetic factors, and the individual's lifestyle (12, 13). The association between lipodystrophy and ART is the most studied and well-established in the literature, especially regarding to protease inhibitors and reverse transcriptase inhibitors (14–17), but some studies described positive cases in which there was no treatment and even so presented changes in body fat composition (18, 19).

The disease's stigma has always been associated with physical appearance and changes in body fat composition indirectly, since the onset of epidemy, weight loss was characterized as one of the main signs associated with HIV infection before the discovering of combined ART. With the ART adverse effects, such as lipodystrophy, new physical evidence of HIV infection emerged, with a negative psychosocial impact, which may negatively influence even treatment adherence (20–22).

With the purpose to define in clinical practice the most appropriate treatment for the prevention of comorbidities and cardiovascular risk reduction, it is important to identify whether the accumulation or distribution of body fat is related to lipid profile and adipocytokines levels, since several changes and interactions are important and related to both the course of the disease and the adverse effects of the specific therapy.

## Materials and Methods

This is a cross-sectional study, in which body composition, metabolic and anthropometric changes, and adipocytokine levels in PLWHA were assessed in a specialized infectiology outpatient service with and without a clinical diagnosis of HALS and a control group. Initially, the PLWHA group with a clinical diagnosis of HALS was defined. From this group, a pairing of sex, age, and body mass index (BMI) was performed for PLWHA group without lipodystrophy and the control group. Thus, the individuals who read and agreed with the free and informed consent form were distributed into groups as follows:

Lipodystrophy group (G1): included 40 individuals with a diagnosis of HIV/AIDS and with a clinical diagnosis of lipodystrophy, on regular ART for at least 1 year of both genders and aged between 27 and 59 years. These individuals joined the study as they presented themselves to the health service for follow-up.

No lipodystrophy group (G2): included 36 individuals diagnosed with HIV/AIDS and without a clinical diagnosis of lipodystrophy, on regular ART for at least 1 year, matched for sex, age, and BMI, representing the experimental group (G1). These individuals joined the study as they presented themselves to the health service for follow-up and the pairing and inclusion criteria were met.

Control group (G3): control group, which included 34 individuals with negative HIV serology, without clinical complaints and matched for age, sex, and BMI, with the experimental group (G1). These individuals were enrolled in the study after checking the inclusion and matching criteria, as they were contacted *via* social media and the University's electronic mail, recruiting subjects with specific gender, age, and BMI.

The study excluded women in the gestational period, individuals with chronic renal disease, nephrotic syndrome, liver disease, uncontrolled hypothyroidism, patients with a pacemaker or any other kind of electronic device in the body, or people who unable to carry out the assessment of body composition. The diagnosis of HALS was made subjectively by the physician, that is, through the detection of specific findings during visual assessment and physical examination performed and informed in the medical record.

Nutritional assessment consisted of measures of weight, height, waist circumference (WC), and neck circumference (NC). Subsequently, the BMI and the waist-to-height ratio (WHtR) were calculated. All anthropometric measurements followed specific methodologies, as explained below.

At first, weight (in kilograms) and height (in meters) were measured. Weight was obtained using a platform-type electronic scale, with a capacity of 150 kg and an accuracy of 0.1 kg. To measure height, a portable anthropometer with 0.1 cm precision was used, with the subject being barefoot and in an orthostatic position. Both measurements were performed following the methodology proposed by Lohman et al. (23). BMI is the measure of body mass in relation to height, calculated using the formula: BMI = weight/height^2^ (kg/m^2^). For the classification of nutritional status, according to BMI value, the values proposed by the World Health Organization (24) were used.

The waist circumference (cm) was evaluated through the abdominal perimeter, being the measurement in place of maximum extension of the abdomen region, according to the methodology recommended by Lohman et al. (23). To perform this measurement, an inextensible metallic measuring tape was used, 2 m long and 0.1 cm accurate.

The NC (cm) was evaluated at the height of the cricothyroid cartilage. In men who had prominence, NC was evaluated below it. For classification, values < 37 cm and > 37 cm for men and < 34 cm and > 34 cm for women were used. The methodology and reference values used were those recommended by Ben-Noun et al. (25). To perform such measurements, an inextensible metallic measuring tape was used, with 2 m length and 0.1 cm precision. The WHtR was obtained by dividing the WC by height (both in centimeters).

The body composition assessment was carried out using a multifrequency bioelectrical impedance (BIA) equipment (Inbody®, model 570 - USA) with a direct segmental evaluation system, being performed with the subject in an orthostatic position and in a room with normal temperature, standing with arms and legs abducted at 45 degrees from the body. The preparation for performing these assessments followed the guidelines of the BIA device manual, which, in general, recommends that the subjects remove all metallic objects from their bodies; be abstinent from alcohol 48 h before the test, do not practice intense physical activity in the 24 h before the test; avoid consuming caffeinated beverages 24 h before the test; fast for at least 2 h; urinate 30 min before the test and rest in the supine position for 8–10 min before the test is performed. Identification, weight, height, age, and gender information were entered directly into the equipment software. After verifying the data, the position of the individual and the electrodes, the exam was started. The measures used were lean mass (kg), fat mass (kg), and total fat percentage (%).

The metabolic profile considered plasma levels of total cholesterol and HDL-cholesterol, triglycerides, and fasting glucose. LDL-cholesterol was calculated using the Friedewald formula (LDL-c = total cholesterol – HDL-cholesterol – triglyceride/5), and non-HDL cholesterol was calculated by subtracting total cholesterol from HDL-cholesterol, being the last analysis performed only for values below 400 mg/dL.

For the assessment of changes in individuals according to the results of the lipid profile, those suggested by the *American College of Cardiology/American Heart Association*, 2013, were used as a reference, total cholesterol ≥ 240 mg/dL, LDL-cholesterol ≥ 160 mg/dL, and triglycerides ≥ 200 mg/ dL; non-HDL cholesterol > 190 and HDL-cholesterol ≥ 40 mg/dL for men and ≥ 50 mg/dL for women. For the classification of hyperglycemia, according to fasting glucose value, the values proposed by the Guidelines of the Brazilian Society of Diabetes, 2019/2020, which suggest reference plasma values ≥100 mg/dL, in fasting, were used.

For the classification of metabolic syndrome (MS), the “harmonized syndrome,” 2009, was used as a reference, defined by the presence of at least three out of five risk factors, increased WC (≥ 90 cm for men and ≥ 80 cm for women), triglycerides ≥ 150 mg/dL, HDL-cholesterol < 40 mg/dL for men and < 50 mg/dL for women, systolic blood pressure ≥ 130 mm Hg or diastolic blood pressure ≥ 85 mm Hg, and fasting glucose > 100 mg/dL.

For the evaluation of cytokines and adipocytokines, 15 ml of peripheral blood was collected by a trained technician. The dosage of the cytokines TNF-α, IFN-γ, IL-6, adiponectin, and leptin was performed using the ELISA technique, using commercial kits (R&D Systems, for human serum samples), according to the manufacturer's instructions.

For statistical treatment, a characterization of the population was initially carried out with descriptive measures calculations (mean, standard deviation, and median) from anthropometry, BIA, and biochemical test data, stratified by group. The comparison between groups was made using the ANOVA test followed by Tukey's multiple comparison test for cases in which data distribution was symmetric. Otherwise, a generalized linear model with gamma distribution followed by Wald's multiple comparison test was used. Comparisons of means were also made following the same techniques for the variables of interest only for women, stratified by age below and above 45 years.

The frequencies of MS by group were obtained, and the association between them was performed using the chi-squared test or Fisher's exact test. Pearson's correlations were calculated for anthropometric, biochemical, and cytokine data for the whole sample and stratified by sex. In all tests, a significance level of 5% or the corresponding *p*-value was set. All analyzes were performed using the SAS software for Windows, v-9.4.

This study was approved by the Research Ethics Committee of Botucatu Medical School, Saõ Paulo State University (CAAE: 26170414.8.0000.5411), and all participants agreed and signed an informed consent form.

## Results

At the end of the study, 110 subjects were assessed, 56 women and 54 men, with a mean age of 45.5 ± 9.3 years. The control group was made up of 34 individuals, the lipodystrophy group by 40 individuals, and the no lipodystrophy group by 36 individuals, and the pairing of sex, age, and BMI was performed by the lipodystrophy group.

The nutritional diagnosis was performed considering the BMI classification, whose average for adults was 25.3 ± 4.5 kg/m^2^, being classified as overweight, according to the WHO, 1995.

When stratifying the total sample by sex (Table 1), it was possible to observe a statistical difference between women for the triglyceride profile, being greater in the group with lipodystrophy compared with the control group (*p* = 0453). The other metabolic parameters showed no statistical difference between women.

**Table 1:** Metabolic profile of women stratified by group of people living with HIV/AIDS with (G1) and without HALS (G2) and control group (G3).

	**G1 (*n* = 20)**	**G2 (*n* = 18)**	**G3 (*n* = 18)**	***p*-value**
Fasting blood glucose (mg/dL)	87.6 ± 14.2	86.2 ± 14.5	85.1 ± 8.7	0.8001
Total cholesterol (mg/dL)	211.9 ± 53.0	198.8 ± 45.2	197.4 ± 35.6	0.5373
HDL-cholesterol (mg/dL)	49.9 ± 14.4	52.4 ± 21.9	49.5 ± 13.0	0.8407
Triglycerides (mg/dL)	204.0 ± 124.8*	158.5 ± 71.8	143.3 ± 43.6	0.0453
LDL-cholesterol (mg/dL)	121.2 ± 42.6	117.0 ±28.1	119.3 ± 25.6	0.9244
Non-HDL cholesterol (mg/dL)	162.0 ± 50.5	146.3 ± 32.2	148.0 ± 30.4	0.4166

*HDL, High-density lipoprotein cholesterol; LDL, Low-density lipoprotein cholesterol; Mg/dL, milligrams per deciliters*.

* *Statistical difference between groups G1 and G3*.

The same statistical difference in triglyceride profile was found in men (Table 2), but unlike women, for men the difference was between the control group and no lipodystrophy group (*p* = 0.0307). The other metabolic parameters showed no statistical difference between men.

**Table 2:** Metabolic profile of men stratified by group of people living with HIV/AIDS with (G1) and without HALS (G2) and control group (G3).

	**G1 (*n* = 20)**	**G2 (*n* = 18)**	**G3 (*n* = 16)**	***p-*value**
Fasting blood glucose (mg/dL)	82.8 ± 10.5	86.9 ± 14.8	85.9 ± 10.2	0.5351
Total cholesterol (mg/dL)	171.9 ± 27.1	178.8 ± 44.7	185.5 ± 48.9	0.5900
HDL-cholesterol (mg/dL)	39.0 ± 14.4	40.7 ± 17.1	46.6 ± 10.0	0.2585
Triglycerides (mg/dL)	190.9 ± 68.1	247.3 ± 222.2*	149.4 ± 83.6	0.0307
LDL-cholesterol (mg/dL)	94.7 ± 26.4	88.6 ± 38.0	109.1 ± 44.9	0.2392
Non-HDL cholesterol (mg/dL)	132.9 ± 31.8	138.1 ± 40.0	138.9 ± 52.1	0.8914

*HDL, High-density lipoprotein cholesterol; LDL, Low-density lipoprotein cholesterol; Mg/dL, milligrams per deciliter*.

* *Statistical difference between G2 and G3 groups*.

Tables 3, 4 represent the body composition between groups stratified into women and men, respectively. There was only one statistical difference for NC variable in men, being this variable greater in the control group compared with the lipodystrophy group (*p* = 0.0162).

**Table 3:** Body composition of women stratified by group of people living with HIV/AIDS with (G1) and without HALS (G2) and control group (G3).

	**G1**	**G2**	**G3**	***p*-value**
BMI (kg/m^2^)	25.8 ± 4.8	25.5 ± 4.8	25.4 ± 5.1	0.9645
PTBF (%)	31.4 ± 8.3	33.1 ± 9.2	34.8 ± 9.0	0.4852
FM (kg)	23.4 ± 9.9	23.1 ± 11.2	23.6 ± 10.4	0.9908
LM (kg)	42.7 ± 8.1	41.0 ± 7.0	39.0 ± 4.9	0.2601
NC (cm)	35.0 ± 4.1	34.0 ± 3.0	34.2 ± 2.9	0.621
AC (cm)	92.0 ± 13.5	90.2 ± 13.35	91.7 ± 11.7	0.9027
WHtR	0.6 ± 0.1	0.6 ± 0.1	0.6 ± 0.1	0.8617

*BMI, body mass index; PTBF, percentage of total body fat; FM, fat mass; LM, lean mass; NC, neck circumference; AC, abdominal circumference; WHtR, waist-to-height ratio*.

**Table 4:** Body composition of men stratified by group of people living with HIV/AIDS with (G1) and without HALS (G2) and control group (G3).

	**G1**	**G2**	**G3**	***p-*value**
BMI (kg/m^2^)	25.3 ± 5.0	24.7 ± 4.1	25.0 ± 3.4	0.9275
PTBF (%)	23.9 ± 9.3	23.3 ± 7.1	2.7 ± 5.5	0.9006
FM (kg)	19.6 ± 10.5	17.5 ± 7.4	18.4 ± 6.7	0.7684
LM (kg)	55.0 ± 9.3	52.0 ± 10.3	57.5 ± 7.9	0.2479
NC (cm)	37.4 ± 3.0	38.0 ± 2.9	40.2 ± 2.8^a^	0.0162
AC (cm)	92.3 ± 14.0	93.1 ± 11.0	95.8 ± 6.8	0.6376
WHtR	0.5 ± 0.1	0.5 ± 0.1	0.5 ± 0.03	0.6969

*BMI, body mass index; PTBF, percentage of total body fat; FM, fat mass; LM, lean mass; NC, neck circumference; AC, abdominal circumference; WHtR, waist-to-height ratio*.

a *Statistical difference between G1 and G3*.

Regarding serum levels of adipocytokines and cytokines, when stratified by sex (Tables 5, 6), a statistically significant difference was found only for TNF-α dosage among men, being higher among subjects without lipodystrophy, compared with men with a diagnosis of HALS (*p* = 0.0222).

**Table 5:** Serum levels of adipocytokines and cytokines in women stratified by the group of people living with HIV/AIDS with (G1) and without HALS (G2) and control group (G3).

	**G1**	**G2**	**G3**	***p*-value**
Adiponectin (μg/mL)	7.3 ± 4.0	8.2 ± 4.3	7.4 ± 7.4	0.8143
Leptin (ng/mL)	14.8 ± 17.8	12.5 ± 11.9	18.2 ± 17.2	0.4958
TNF-α (pg/mL)	10.8 ± 14.4	6.5 ± 3.4	10.2 ± 18.2	0.139
IFN-γ (pg/mL)	6.2 ± 16.5	2.5 ± 2.6	5.2 ± 16.5	0.1848
IL-6 (pg/mL)	4.1 ± 6.1	2.1 ± 2.0	3.5 ± 3.9	0.1493

*TNF-α, tumoral necrosis factor alfa; IFN-γ, interferon gamma; IL-6, interleukin 6; μg/mL, micrograms per milliliter; ng/mL, nanograms per milliliter; pg/mL, picograms per milliliter*.

**Table 6:** Serum levels of adipocytokines and cytokines in men stratified by a group of people living with HIV/AIDS with (G1) and without HALS (G2) and control group (G3).

	**G1**	**G2**	**G3**	***p*-value**
Adiponectin (μg/mL)	6.4 ± 4.8	8.8 ± 6.0	8.2 ± 6.9	0.3412
Leptin (ng/mL)	20.8 ± 17.5	19.4 ± 15.1	29.0 ± 51.1	0.3372
TNF-α (pg/mL)	10.4 ± 14.3^a^	25.4 ± 44.2	13.0 ± 28.2	**0.0222**
IFN-γ (pg/mL)	8.7 ± 13.1	7.6 ± 24.4	6.8 ± 22.7	0.6870
IL-6 (pg/mL)	5.2 ± 6.0	3.7 ± 4.8	3.8 ± 4.0	0.4980

*TNF-α, tumoral necrosis factor alfa; IFN-γ, interferon gamma; IL-6, interleukin 6; μg/mL, micrograms per milliliter; ng/mL, nanograms per milliliter; pg/mL, picograms per milliliter*.

a *Statistical difference between groups G1 and G2*.

*The bold values highlight the statistical significance (p < 0.05)*.

Table 7 shows the comparisons of metabolic nutritional parameters and cytokines between women under and over 45 years old. Statistical difference was found for anthropometric parameters related to fat accumulation: BMI (*p* = 0.0007), PTBF (*p* = 0.0009), NC (*p* = 0.0094), WC (*p* = 0.0023), and WHtR (*p* = 0.0016) were higher in women under 45 years old. Regarding serum levels of cytokines and adipocytokines, a statistical difference was found for the mean value of adiponectin, which was also higher in women under 45 years old (*p* = 0.0031). No differences were observed for metabolic profile.

**Table 7:** Comparative values between women below and above 45 years old for anthropometric parameters, body composition, adipocytokines, and cytokines.

**Variable**	**Age ≤ 45 years**	**Age > 45 years**	***p*-value**
BMI (Kg/m^2^)	27.5 ± 4.5	23.2 ± 4.2^$^	**0.0007**
PTBF (%)	36.4 ± 7.8	28.9 ± 8.2^$^	**0.0009**
MM (Kg)	42.4 ± 6.8	39.2 ± 6.7	0.0849
NC (cm)	35.5 ± 3.0	33.1 ± 3.5^$^	**0.0094**
WC (cm)	95.9 ± 12.5	85.7 ± 10.7^$^	**0.0023**
WHtR	0.6 ± 0.07	0.5 ± 0.1^$^	**0.0016**
ADIPO (μg/mL)	9.2 ± 6.14	5.7 ± 3.3^$^	**0.0031**
LEP (ng/mL)	1.7 ± 1.61	1.3 ± 1.6	0.3376
TNF-α (pg/mL)	9.8 ± 18.0	8.6 ± 3.6	0.5021
IFN-γ (pg/mL)	6.0 ± 18.0	3.1 ± 2.5^$^	**0.0016**
IL-6 (pg/mL)	3.5 ± 4.7	3.0 ± 4.1	0.8747
FG (mg/dL)	86.0 ± 12.8	86.8 ± 11.1	0.8187
TC (mg/dL)	201.1 ± 43.6	205.4 ± 47.9	0.7179
HDL (mg/dL)	51.2 ± 14.4	49.8 ± 19.1	0.7584
TGL (mg/dL)	162.9 ± 87.7	178.5 ± 95.9	0.4653
LDL (mg/dL)	117.3 ± 31.2	121.6 ± 35.2	0.6238
Non-HDL-C (mg/dL)	149.9 ± 42.2	155.6 ± 39.8	0.6

*BMI, body mass index; PTBF, percentage of total body fat; FM, fat mass; LM, lean mass; NC, neck circumference; AC, abdominal circumference; WHtR, waist-to-height ratio; Adipo, adiponectin; Lep, leptin; TNF-α, tumoral necrosis factor alfa; IFN-γ, interferon gamma; IL-6, interleukin 6; GJ, fasting glucose; CT, total cholesterol; HDL, high-density lipoprotein cholesterol; TGL, triglycerides; LDL, low-density lipoprotein cholesterol; non-HDL-C, non-HDL cholesterol; μg/mL, micrograms per milliliter; ng/mL, nanograms per milliliter; pg/mL, picograms per milliliter*.

$ *Difference between women below and above 45 years old with p < 0.05*.

*The bold values highlight the statistical significance (p < 0.05)*.

Table 8 shows the comparison between male and female subjects within groups for anthropometric parameters, body composition, adipocytokines, and cytokines. For G1 group, significantly higher values of LM (*p* = < 0.0001) and CP (*p* = 0.0458) were found in male subjects. Significantly higher values of TC (*p* = 0.0035), HDL (*p* = 0.0184), LDL (*p* = 0.0193), and non-HDL-C (*p* = 0.0262) were found for female subjects.

**Table 8:** Comparison between men and women intragroup of people living with HIV/AIDS with (G1) and without HALS (G2) and control group (G3), for anthropometric, body composition, and adipocytokine and cytokine parameters.

**Variável**	**G1**			**G2**			**G3**		
	**Men**	**Women**	***p*-value**	**Men**	**Women**	***p*-value**	**Men**	**Women**	***p*-value**
BMI (kg/m^2^)	25.3 ± 5.0	25.8 ± 4.8	0.7268	24.7 ± 4.1	25.5 ± 4.9	0.6056	25.0 ± 3.36	25.4 ± 5.2	0.796
PTBF (%)	23.9 ± 9.3	31.4 ± 8.3	0.0108	23.3 ± 7.1	33.1 ± 9.2^$^	0.0013	22.7 ± 5.46	34.8 ± 9.0*	<0.0001
MM (kg)	55.0 ± 9.3	42.7 ± 8.1*	<0.0001	52.0 ± 10.3	41.0 ± 7.0^$^	0.0007	57.5 ± 7.86	39.0 ± 4.9*	<0.0001
NC (cm)	37.4 ± 3.0	35.0 ± 4.1^$^	0.0458	38.0 ± 2.9	34.0 ± 3.0^$^	0.0003	40.2 ± 2.78	34.2 ± 2.9*	<0.0001
WC (cm)	92.3 ± 13.9	92.0 ± 13.5	0.9498	93.1 ± 11.0	90.2 ± 13.3	0.4889	95.8 ± 6.81	91.7 ± 11.7	0.2291
WHtR	0.5 ± 0.1	0.6 ± 0.1	0.1087	0.5 ± 0.1	0.6 ± 0.1	0.4727	0.5 ± 0.03	0.6 ± 0.1	0.0876
Adipo (μg/mL)	6.4 ± 4.8	7.3 ± 4.0	0.4847	8.8 ± 6.0	8.3 ± 4.3	0.7762	8.3 ± 6.92	7.4 ± 7.4	0.6855
Lel (ng/mL)	2.1 ± 1.8	1.5 ± 1.8	0.2464	1.9 ± 1.5	1.2 ± 1.2	0.1318	2.9 ± 2.11	1.8 ± 1.8	0.1413
TNF-α (pg/mL)	10.4 ± 14.3	10.8 ± 14.4	0.8781	25.4 ± 44.2	6.5 ± 3.4*	<0.0001	13.0 ± 28.2	10.2 ± 18.2	0.6356
IFN-γ (pg/mL)	8.7 ± 13.1	6.2 ± 16.5	0.2383	7.6 ± 24.4	2.5 ± 2.6	0.1081	6.8 ± 22.7	5.2 ± 16.5	0.7095
IL-6 (pg/mL)	5.2 ± 6.0	4.1 ± 6.1	0.6146	3.7 ± 4.8	2.1 ± 2.0	0.0739	3.8 ± 4.0	3.5 ± 3.9	0.9491
FG (mg/dL)	82.8 ± 10.5	87.7 ± 14.2	0.2122	86.9 ± 14.8	86.2 ± 12.5	0.8708	85.9 ± 10.2	85.1 ± 8.7	0.8074
TC (mg/dL)	171.9 ± 27.1	211.9 ± 5^$^	0.0035	178.8 ± 44.7	198.8 ± 45.2	0.175	185.5 ± 48.9	197.4 ± 35.6	0.4001
HDL (mg/dL)	39.0 ± 14.5	49.9 ± 14.4^$^	0.0184	40.7 ± 17.1	52.4 ± 21.9	0.0718	46.6 ± 10.0	49.5 ± 13.0	0.4522
TGL (mg/dL)	190.9 ± 68.1	204.0 ± 124.8	0.6474	247.3 ± 222.2	158.5 ± 71.9^$^	0.0318	149.4 ± 83.6	143.3 ± 43.6	0.7562
LDL (mg/dL)	94.7 ± 26.4	121.2 ± 42.6^$^	0.0193	88.6 ± 38.0	117.0 ± 28.1^$^	0.012	109.0 ± 44.9	119.3 ± 25.6	0.3963
Non-HDL-C (mg/dL)	132.9 ± 31.8	162.0 ± 50.5^$^	0.0262	138.1 ± 39.8	146.3 ± 38.2	0.5187	138.9 ± 52.7	147.9 ± 30.4	0.556

*BMI, body mass index; PTBF, percentage of total body fat; FM, fat mass; LM, lean mass; NC, neck circumference; AC, abdominal circumference; WHtR, waist-to-height ratio; Adipo, adiponectin; Lep, leptin; TNF-α, tumoral necrosis factor alfa; IFN-γ, interferon gamma; IL-6, interleukin 6; GJ, fasting glucose; CT, total cholesterol; HDL, high-density lipoprotein cholesterol; TGL, triglycerides; LDL, low-density lipoprotein cholesterol; non-HDL-C, non-HDL cholesterol; μg/mL, micrograms per milliliter; ng/mL, nanograms per milliliter; pg/mL, picograms per milliliter*.

* *Difference between men and women in each group with p < 0.0001*;

$ *Difference between men and women in each group with p < 0.05*.

Table 9 shows the results of the correlations between anthropometric indicators, body composition, adipocytokines, and cytokines for the general sample. WHtR collinearity was verified with all anthropometric parameters, except muscle mass. A strong association between IFN-γ and TNF-α (*r* = 0.97; *p* = < 0.0001) was found. Weak correlations between serum leptin levels with muscle mass (*r* = 0.24; *p* = 0.0105) and NC (*r* = 0.27; *p* = 0.0046) were found.

**Table 9:** Simple Pearson's correlation coefficients (r) for the total sample between anthropometric parameters, body composition, adipocytokines, and cytokines.

	**BMI**	**PTBF**	**MM**	**NC**	**WC**	**WHtR**	**Adipo**	**Lep**	**TNF-α**	**IFN-γ**	**IL-6**	**FG**	**TC**	**HDL**	**TGL**	**LDL**	**Non-HDL-C**
BMI	1.00	**0.69**	**0.45**	**0.58**	**0.82**	**0.78**	0.05	0.08	0.00	0.13	0.14	0.06	−0.06	**−0.29**	0.08	−0.01	0.04
PTBF		1.00	**−0.23**	0.09	**0.54**	**0.73**	0.054	−0.06	−0.06	0.05	0.03	−0.05	0.18	−0.05	0.08	0.18	0.21
MM			1.00	**0.74**	**0.48**	0.08	0.01	0.24	0.07	0.11	0.21	0.01	−0.28	**−0.36**	0.02	−0.20	−0.15
NC				1.00	**0.72**	**0.45**	0.07	0.27	0.11	0.17	0.18	0.02	−0.20	−0.33	−0.03	−0.09	−0.09
WC					1.00	**0.90**	0.08	0.18	0.08	0.20	0.23	0.10	−0.06	−0.29	0.03	0.03	0.04
WHtR						1.00	0.06	0.04	0.04	0.17	0.16	0.10	0.06	−0.16	0.04	0.12	0.13
Adipo							1.00	−0.03	0.07	0.02	0.08	−0.08	0.07	−0.04	−0.04	0.12	0.09
Lep								1.00	0.00	0.02	0.06	0.22	−0.03	−0.03	0.14	−0.13	−0.03
TNF-α									1.00	**0.86**	**0.53**	−0.01	−0.16	−0.07	−0.04	−0.14	−0.14
IFN-γ										1.00	**0.58**	−0.05	−0.14	−0.08	−0.06	−0.10	−0.12
IL−6											1.00	−0.02	−0.13	−0.10	−0.13	−0.03	−0.10
FG												1.00	−0.04	−0.05	0.09	−0.10	−0.02
TC													1.00	**0.36**	0.35	**0.81**	**0.93**
HDL														1.00	−0.27	0.15	−0.01
TGL															1.00	−0.12	**0.48**
LDL																1.00	**0.81**
Non-HDL-C																	1.00

*BMI, body mass index; PTBF, percentage of total body fat; FM, fat mass; LM, lean mass; NC, neck circumference; AC, abdominal circumference; WHtR, waist-to-height ratio; Adipo, adiponectin; Lep, leptin; TNF-α, tumoral necrosis factor alfa; IFN-γ, interferon gamma; IL-6, interleukin 6; GJ, fasting glucose; CT, total cholesterol; HDL, high-density lipoprotein cholesterol; TGL, triglycerides; LDL, low-density lipoprotein cholesterol; non-HDL-C, non-HDL cholesterol*.

*Bold values present p < 0.0001; underlined values present p < 0.05*.

Table 10 shows the results of the correlations between anthropometric indicators, body composition, adipocytokines, and cytokines for women. Collinearity was verified among the WHtR with all anthropometric parameters. A strong association between IFN-γ and TNF-α (*r* = 0.97; *p* = < 0.0001) was found. Moderate correlations were identified between leptin and GJ (*r* = 0.42; *p* = 0.0012) and between IL-6 and TNF-α (*r* = 0.56; *p* = < 0.0001) and IFN-γ (*r* = 0.57; *p* = < 0.0001).

**Table 10:** Simple Pearson's correlation coefficients (r) among women for anthropometric parameters, body composition, adipocytokines, and cytokines.

	**BMI**	**PTBF**	**MM**	**NC**	**WC**	**WHtR**	**Adipo**	**Lep**	**TNF-α**	**IFN-γ**	**IL-6**	**FG**	**TC**	**HDL**	**TGL**	**LDL**	**Non-HDL-C**
BMI	1.00	**0.77**	**0.66**	**0.80**	**0.87**	**0.83**	0.11	0.08	0.26	0.31	0.24	0.18	−0.14	−0.27	−0.04	−0.05	−0.04
PTBF		1.00	0.21	**0.56**	**0.73**	**0.77**	0.10	0.16	0.16	0.20	0.14	−0.02	0.07	−0.10	0.06	0.10	0.12
MM			1.00	**0.68**	**0.57**	0.37	0.04	0.07	0.14	0.18	0.23	0.25	−0.25	−0.34	−0.11	−0.12	−0.14
NC				1.00	**0.82**	**0.75**	0.01	0.12	0.20	0.24	0.16	0.22	−0.20	−0.37	−0.08	−0.05	−0.06
WC					1.00	**0.96**	0.08	0.14	0.25	0.32	0.27	0.21	−0.14	−0.27	−0.05	−0.04	−0.04
WHtR						1.00	0.08	0.05	0.27	0.33	0.26	0.14	−0.08	−0.21	−0.03	0.00	0.00
Adipo							1.00	−0.11	−0.12	−0.12	0.17	−0.02	0.00	0.07	−0.05	−0.03	−0.03
Lep								1.00	0.20	0.20	0.04	0.42	0.01	0.07	0.13	−0.11	−0.01
TNF-α									1.00	**0.97**	**0.56**	0.18	0.02	−0.05	0.03	0.04	0.05
IFN–γ										1.00	**0.57**	0.20	−0.02	−0.08	0.03	0.00	0.01
IL−6											1.00	0.13	0.03	−0.09	−0.05	0.11	0.08
FG												1.00	0.02	−0.13	0.24	−0.07	0.08
TC													1.00	0.43	0.47	**0.85**	**0.93**
HDL														1.00	−0.17	0.14	0.07
TGL															1.00	0.17	**0.59**
LDL																1.00	**0.88**
Non-HDL-C																	1.00

*BMI, body mass index; PTBF, percentage of total body fat; FM, fat mass; LM, lean mass; NC, neck circumference; AC, abdominal circumference; WHtR, waist-to-height ratio; Adipo, adiponectin; Lep, leptin; TNF-α, tumoral necrosis factor alfa; IFN-γ, interferon gamma; IL-6, interleukin 6; GJ, fasting glucose; CT, total cholesterol; HDL, high-density lipoprotein cholesterol; TGL, triglycerides; LDL, low-density lipoprotein cholesterol; non-HDL-C, non-HDL cholesterol*.

*Bold values present p < 0.0001; underlined values present p < 0.05*.

Table 11 shows the results of correlations between anthropometric indicators, body composition, adipocytokines, and cytokines for men. WHtR collinearity was verified with all anthropometric parameters, except muscle mass. A strong association between IFN-γ and TNF-α was identified (*r* = 085; *p* = < 0.0001). Moderate associations between IL-6 with TNF-α (*r* = 0.55; *p* = < 0.0001) and IFN-γ (*r* = 0.59; *p* = < 0.0001) were found.

**Table 11:** Simple Pearson's correlation coefficients (r) among men for anthropometric parameters, body composition, adipocytokines, and cytokines.

	**BMI**	**PTBF**	**MM**	**NC**	**WC**	**WHtR**	**Adipo**	**Lep**	**TNF-α**	**IFN-γ**	**IL-6**	**FG**	**TC**	**HDL**	**TGL**	**LDL**	**Non-HDL-C**
BMI	1.00	**0.74**	**0.67**	**0.61**	**0.78**	**0.71**	−0.02	0.11	−0.13	0.00	0.06	−0.09	−0.01	−0.37	0.19	−0.02	0.12
PTBF		1.00	0.11	0.42	**0.62**	**0.68**	0.00	−0.04	−0.05	0.05	0.06	−0.17	0.00	−0.41	0.27	−0.05	0.14
MM			1.00	**0.58**	**0.55**	0.25	0.01	0.20	−0.10	0.01	0.15	−0.08	−0.04	−0.19	−0.06	0.08	0.03
NC				1.00	**0.75**	**0.61**	0.16	0.25	−0.03	0.07	0.14	−0.12	0.10	−0.04	−0.14	0.23	0.11
WC					1.00	**0.93**	0.09	0.18	−0.02	0.10	0.17	−0.02	0.09	−0.29	0.06	0.16	0.19
WHtR						1.00	0.05	0.14	−0.01	0.09	0.11	0.03	0.11	−0.27	0.15	0.11	0.20
Adipo							1.00	0.03	0.15	0.10	0.01	−0.14	0.16	−0.16	−0.03	0.26	0.21
Lep								1.00	−0.12	−0.12	0.04	0.06	0.05	0.00	0.11	−0.04	0.05
TNF–α									1.00	**0.85**	**0.55**	−0.08	−0.21	−0.02	−0.08	−0.16	−0.20
IFN–γ										1.00	**0.59**	−0.22	−0.21	−0.04	−0.12	−0.12	−0.20
IL-6											1.00	−0.15	−0.25	−0.05	−0.20	−0.09	−0.22
FG												1.00	−0.15	0.02	0.01	−0.18	−0.15
TC													1.00	0.13	0.38	**0.74**	**0.94**
HDL														1.00	−0.33	0.01	−0.22
TGL															1.00	−0.25	0.49
LDL																1.00	**0.72**
Non-HDL-C																	1.00

*BMI, body mass index; PTBF, percentage of total body fat; FM, fat mass; LM, lean mass; NC, neck circumference; AC, abdominal circumference; WHtR, waist-to-height ratio; Adipo, adiponectin; Lep, leptin; TNF-α, tumoral necrosis factor alfa; IFN-γ, interferon gamma; IL-6, interleukin 6; GJ, fasting glucose; CT, total cholesterol; HDL, high-density lipoprotein cholesterol; TGL, triglycerides; LDL, low-density lipoprotein cholesterol; non-HDL-C, non-HDL cholesterol*.

*Bold values present p < 0.0001; underlined values present p < 0.05*.

## Discussion

Understanding the endocrine role of adipose tissue and its alterations in PLWHA, and also the metabolic nutritional profile of these subjects, becomes essential, as they can be important determinants of cardiovascular risk, especially for those with lipodystrophy syndrome changes. Adipose tissue plays, in addition to its role in lipid storage, a crucial function for insulin sensitivity, in addition to modulating, through the secretion of adipocytokines, the metabolism of glucose and lipids.

Metabolic syndrome represents a set of biochemical and physiological risk factors that are directly associated with increased risk for cardiovascular disease and type 2 diabetes. The prevalence has increased exponentially worldwide, as has obesity, a public health crisis. The prevalence of MS in developing countries has increased quickly, mostly due to changes in lifestyle driven by industrialization (26). Reports from South America estimated in 2011 a prevalence of 33% in Brazil (27).

In this study, MS was identified in half of the male sample for both groups of PLWHA with (*n* = 10) and without clinical diagnosis (*n* = 9) of lipodystrophy, and in 18.75% (*n* = 3) for the group control. For women, similar values were found for PLWHA without (50%, *n* = 9) and with (55%, *n* = 11) clinical diagnosis of lipodystrophy and a third of the control group (*n* = 6). There was no positive association between groups when the sample was stratified by female (*p* = 0.3821) and male (*p* = 0.1026) subjects.

In individuals with lipodystrophy, there may be a limitation in the storage of triglycerides in the adipose tissue, which results in the relocation of triglycerides to non-typical sites, the liver or skeletal muscles, and development of insulin resistance (18, 19). The present research identified higher serum triglyceride values both in women with lipodystrophy (*p* = 0.0453) and in men living with HIV/AIDS (*p* = 0.0307) in relation to the control group, when stratified by sex. When stratified by group, no significant difference in lipid profile between genders in the control group was found. For PLWHA group, it was identified a higher mean value of triglycerides for men (*p* = 0.0318) and a higher mean value of LDL for women (*p* = 0.012). The greatest differences were observed in the group of PLWHA with a clinical diagnosis of HALS, being the values of total cholesterol (*p* = 0.0035), HDL-cholesterol (*p* = 0.0184), LDL-cholesterol (*p* = 0.0193), and non-HDL cholesterol (*p* = 0.0268) higher for female subjects. Another study that also assessed the lipid profile between genders found a statistical difference only for triglyceride levels (28). It is noteworthy that women have higher values of HDL-cholesterol compared to men. In addition to the HIV infection itself also playing a role in lipid alterations (29), such disorders are also associated with changes and accumulation of body fat (30). It is important to emphasize that the mean BMI of those women living with HIV/AIDS was 25.81 ± 4.77 kg/m^2^ and the total percentage of body fat was 31.36 ± 8.26% (23.16 ± 9.89 kg), being considered as excessive.

There are reports in the literature of the relationship between estrogen and body fat, through the reduction in the activity of abdominal lipolysis. Women in the climacteric or menopause phase, a period in which there is a reduction in estrogen, have an increase in fat mass and redistribution of body fat to the abdominal area, a risk factor for developing cardiovascular events (31, 32). Since no difference for WC, neck, or WHtR between sexes or groups was found, to investigate differences between women considering the climacteric period or menopause, these subjects were stratified as below or above 45 years. Interestingly, anthropometric values showed higher variations for women under 45 years, including the mean value of adiponectin (*p* = 0.0031). No differences were observed for metabolic profile.

Adipose tissue is an important focus of inflammation in the context of obesity, with recruitment of macrophages to this site, as these cells produce proinflammatory cytokines. In the context of HIV infection, adipose tissue is a crucial cofactor both for viral persistence and for activation and chronic immune inflammation with cardiovascular and metabolic consequences. Adipocytes from obese individuals express disproportionately high levels of TNF-α, characteristic of a systemic inflammatory response (33), while free serum levels are strongly correlated with BMI (34). In the present, it was possible to observe higher serum levels of TNF-α in men living with HIV/AIDS without lipodystrophy, compared with men with HALS. Opposing such findings, the literature reports an increase in the expression of IL-6 and TNF-α in PLWHA diagnosed with lipoatrophy (35).

When stratifying by sex, it was possible to observe a statistical difference between PLWHA group without HALS, with serum levels of TNF-α being higher in men (*p* = < 0.0001). Positive correlations between such variables with IFN-γ (*r* = 0.86, *p* = < 0.0001) and IL-6 (*r* = 0.53, *p* = < 0.0001) were observed. No evaluation considering the method and treatment period to better elucidate these findings among PLWHA groups was conducted, but these data may better reflect the inflammatory status of those individuals, since both exposure to the virus and different treatment methods have a direct relationship to the disease's chronicity.

Interferons are a class of proteins involved in natural defense, which act by inducing resistance to viral infections and modulating the immune response (36). Increased plasma concentrations of IFN-γ have been documented in several viral infections, including HIV (37). In this study, it was not possible to observe differences in serum levels of this variable between groups, but we found a positive correlation with IL-6 levels (*r* = 0.59, *p* = < 0.0001).

In parallel with the antiviral activity, IFN-γ has considerable endocrine and metabolic effects, mainly by regulating lipid metabolism, as evidenced by the following observations: it stimulates hepatic lipogenesis, increasing hepatic *de novo* fatty acid synthesis and VLDL production; reducing the activity of lipoprotein lipase in adipose tissue; increases lipolysis through a direct effect on fat cells; and decreasing triglyceride clearance (38). Despite the findings in the literature, it was impossible to observe a correlation between lipid profile and IFN-γ levels in the present research.

Recent studies have shown that plasma levels of adiponectin and IL-6 are inversely correlated, suggesting that cytokines are possible regulators of adiponectin production (39, 40), but it was not possible to identify such an association in this research. Cross-sectional studies showed that acute phase reactants such as C-reactive protein (CRP) and cytokines (lL-6 and TNF-α) are related to components of the MS, such as BMI, WC, resistance to insulin, hypertension, and dyslipidemia (41–44). This study identified a positive correlation between serum IL-6 levels with weight (*r* = 0.21, *p* = 0.0315) and lean mass (*r* = 0.21, *p* = 0.0282). Such outcome was also found between abdominal obesity and circulating levels of IL-6 in another study (45). The present data indicate that WC had a positive relationship with both IFN-γ (*r* = 0.20, *p* = 0.0373) and IL-6 (*r* = 0.23, *p* = 0.0162).

Adiponectin is an adipocyte-specific cytokine, previously observed as abnormal in the context of HIV infection and lipodystrophy (46). Such protein is known to have metabolic effects that include increased insulin sensitivity and potential cardioprotective effects in the general population (47, 48). In the present research, it was not possible to observe differences in serum adipocytokine levels between groups, and also positive correlations with other parameters. Tong et al. (49), who also assessed the same matched by sex and BMI groups, also found no difference between serum adiponectin levels between groups, but an inverse correlation between adiponectin levels and HALS (49) reduced in individuals with fat redistribution, suggesting that such alteration may contribute to the development of lipodystrophy syndrome (46, 48). It is important to note that the aforementioned study only assessed male individuals with a mean BMI of 24.8 kg/m^2^, that is, eutrophic. In the present research, even stratified by sex, no difference between serum adiponectin levels among men from different groups was evidenced, although the BMI was 25.3 kg/m^2^, considered as overweight. Furthermore, there was no statistical difference for both sexes between the total fat percentage of the groups, which suggests a homogeneity between them in relation to the total amount of fat mass, failing to agree with the literature, which shows a different fat percentage between groups. Another point of consideration is the lipodystrophy classification, which in this study was performed according to the clinical diagnosis, that is, from the agreement between the individual's complaints and the clinician's observations, while in other comparative studies, in addition to the clinical complaint, the waist–hip ratio was also used as one of the defining parameters of lipodystrophy.

As a hormone secreted by adipocytes, leptin primarily reflects the fat mass, acting as an important regulator of energy homeostasis (50). Like adiponectin, leptin is expected to be reduced in PLWHA with HALS (51), but it was not possible to observe any difference in serum leptin levels between groups in the present investigation, even when stratified by sex. It is noteworthy that the lack of statistical difference is due to a high standard deviation for all groups, since observing the brute mean values, it is possible to notice a trend of reduced leptin values in PLWHA compared with the control group, but, again, with no statistical relevance. On the other hand, positive correlations with weight (*r* = 0.31, *p* = 0.0395), lean mass (*r* = 0.24, *p* = 0.0105), NC (*r* = 0.27, *p* = 0.0046), and fasting glucose (*r* = 0.21, *p* = 0.0234) were observed. Vigouroux et al. (51) also found a positive correlation between serum levels of leptin and fasting glucose (52).

The profile of PLWHA has changed with the advancement of disease-specific therapy, going from an acute disease to a chronic disease controlled by ART, revealing other health disturbances, in addition to limnological changes. These factors contribute to the development of non-AIDS-related comorbidities and the main causes of death in PLWHA are associated with cardiovascular diseases, diabetes mellitus, dyslipidemia, neurological disorders, kidney and liver diseases, bone disorders, and non-AIDS-related cancers. If before caring about this group was related to the wasting syndrome, today the main concern is in relation to changes in nutritional status, related to excessive weight and consequent metabolic alterations. Thus, it is essential to trace the metabolic nutritional profile of these individuals to seek interventions and improve the health and life quality of PLWHA, trying to reduce both the impact of the infection and the adverse effects of ART. In the DAD (53) study (Data Collection on Adverse Events of Anti-HIV Drugs), a large multinational prospective cohort study of adverse events in PLWHA, the authors showed a strong association of increased period of ART administration with higher rates of myocardial infarction. The authors highlight the importance of studying the relationship between metabolic disorders associated with ART and their contribution to the premature development of cardiovascular diseases, since such findings directly imply the clinical treatment of the illness, with the emphasis on non-pharmaceutical measures aimed at reducing cardiovascular disease risk (54), correlations that should be investigated in future study.

As a clinical study, limitations from individuals' recruitment to the literature outcome comparison are challenges to be mitigated, since several factors can interfere in this process. Although the data investigated and the results achieved indicate a substantial advance in this field of knowledge, more in-depth studies with longitudinal approaches could further elucidate the implications of the cytokine and metabolic nutritional profile of PLWHA, mainly related to metabolic and lipodystrophy syndromes.

## Conclusion

Collinearity of WHtR with all anthropometric parameters was identified both for the general sample and for gender, except for muscle mass. A strong association between IFN-γ and TNF-α was identified both for the general sample and for gender, and a moderate correlation between leptin and fasting glucose for women was showed.

Decrease in triglyceride profile was observed both for women with lipodystrophy and for men in PLWHA without lipodystrophy when compared to each respective control group. Higher serum TNF-α values were found among men without lipodystrophy compared to those with HALS.

Considering the syndrome's manifestations, these patients have a high-risk endocrine metabolic profile for cardiovascular events. Due to this fact, it is necessary to invest in strategies aimed at preventing and controlling possible comorbidities to improve patient survival.

## Data Availability Statement

The original contributions presented in the study are included in the article/supplementary material, further inquiries can be directed to the corresponding author/s.

## Ethics Statement

The studies involving human participants were reviewed and approved by Human Research Ethics Committee of São Paulo State University (Unesp), Medical School, Botucatu. The patients/participants provided their written informed consent to participate in this study.

## Author Contributions

LBS contributed to the design and implementation of the research, to the analysis of the results, and to the writing of the manuscript. SJP and JS were involved in planning and supervised the work. ALM supervised the findings of this work. MG carried out the experiment. PCMP conceived the original idea. JEC processed the experimental data and performed the analysis. All authors contributed to the article and approved the submitted version.

## Funding

This study was financed in part by the Coordenação de Aperfeiçoamento de Pessoal de Nível Superior—Brasil (CAPES)—finance code 001; grant 2015/10103-7, São Paulo Research Foundation (FAPESP).

## Conflict of Interest

The authors declare that the research was conducted in the absence of any commercial or financial relationships that could be construed as a potential conflict of interest.

## Publisher's Note

All claims expressed in this article are solely those of the authors and do not necessarily represent those of their affiliated organizations, or those of the publisher, the editors and the reviewers. Any product that may be evaluated in this article, or claim that may be made by its manufacturer, is not guaranteed or endorsed by the publisher.

## References

[1] Teeraananchai S Kerr S Amin J Ruxrungtham K Law M. Life expectancy of HIV-positive people after starting combination antiretroviral therapy: a meta-analysis. HIV Med. (2017) 18:256–66. 10.1111/hiv.12421 27578404

[2] Katz IT Maughan-Brown B. Improved life expectancy of people living with HIV: who is left behind? Lancet HIV. (2017) 4:e324–e6. 10.1016/S2352-3018(17)30086-3 28501496 PMC5828160

[3] Urias E. The potential synergies between industrial and health policies for access to medicines: insights from the Brazilian policy of universal access to HIV/AIDS treatment. Innovation Dev. (2019) 9:245–60. 10.1080/2157930X.2019.1567964

[4] Carr A Cooper DA. Adverse effects of antiretroviral therapy. Lancet. (2000) 356:1423–30. 10.1016/S0140-6736(00)02854-3 11052597

[5] Alves MD Brites C Sprinz E. HIV-associated lipodystrophy: a review from a Brazilian perspective. Therap Clin Risk Manage Auckland. (2014) 10:559–66. 10.2147/TCRM.S35075 25083134 PMC4108257

[6] Luz PM Veloso VG Grinsztejn B. The HIV epidemic in Latin America: accomplishments and challenges on treatment and prevention. Curr Opin HIV AIDS. (2019) 14:366. 10.1097/COH.0000000000000564 31219888 PMC6688714

[7] Benzaken AS Pereira GF Costa L Tanuri A Santos AF Soares MA. Antiretroviral treatment, government policy and economy of HIV/AIDS in Brazil: is it time for HIV cure in the country? AIDS Res Therapy. (2019) 16:1–7. 10.1186/s12981-019-0234-2 31412889 PMC6694665

[8] Bedimo RJ. Body-fat abnormalities in patients with HIV: progress and challenges. J Int Assoc Phys AIDS Care. (2008) 7:292–305. 10.1177/1545109708328931 19056708

[9] Cunha CLPd. Lipodystrophy Associated with HIV/ART and Cardiovascular Risk Factors. SciELO Brasil (2020). 10.36660/ijcs.20200302

[10] Guzman N Vijayan V. HIV-associated Lipodystrophy. In: StatPearls [Internet]. Treasure Island, FL: StatPearls Publishing (2021) Available online at: https://www.ncbi.nlm.nih.gov/books/NBK493183/ (accessed May 12, 2021).

[11] Lagathu C Béréziat V Gorwood J Fellahi S Bastard J-P Vigouroux C. Metabolic complications affecting adipose tissue, lipid and glucose metabolism associated with HIV antiretroviral treatment. Exp Opin Drug Safe. (2019) 18:829–40. 10.1080/14740338.2019.1644317 31304808

[12] Martinez E Mocroft A García-Viejo MA Pérez-Cuevas JB Blanco JL Mallolas J. Risk of lipodystrophy in HIV-1-infected patients treated with protease inhibitors: a prospective cohort study. Lancet. (2001) 357:592–8. 10.1016/S0140-6736(00)04056-3 11558485

[13] Batterham MJ Garsia R Greenop PA. Dietary intake, serum lipids, insulin resistance and body composition in the era of highly active antiretroviral therapy 'Diet FRS Study'. Aids. (2000) 14:1839–43. 10.1097/00002030-200008180-00020 10985322

[14] Villarroya F Domingo P Giralt M. Lipodystrophy associated with highly active anti-retroviral therapy for HIV infection: the adipocyte as a target of anti-retroviral-induced mitochondrial toxicity. Trends Pharmacol Sci. (2005) 26:88–93. 10.1016/j.tips.2004.12.005 15681026

[15] Carr A Samaras K Chisholm DJ Cooper DA. Pathogenesis of HIV-1-protease inhibitor-associated peripheral lipodystrophy, hyperlipidaemia, and insulin resistance. Lancet. (1998) 351:1881–3. 10.1016/S0140-6736(98)03391-1 9652687

[16] Koethe JR Lagathu C Lake JE Domingo P Calmy A Falutz J. HIV and antiretroviral therapy-related fat alterations. Nat Rev Dis Primers. (2020) 6:1–20. 10.1038/s41572-020-0181-1 32555389

[17] Nzuza S Zondi S Hurchund R Owira PM. Highly active antiretroviral therapy-associated metabolic syndrome and lipodystrophy: pathophysiology and current therapeutic interventions. J Endocrinol Metab. (2017) 7:103–16. 10.14740/jem364w

[18] Alencastro PR Wolff FH Schuelter-Trevisol F Ikeda ML Brandão ABdM Barcellos NT. Characteristics associated to lipodystrophy syndrome among HIV infected patients naive and on antiretroviral treatment. J AIDS Clin Res. (2012) 3:182. 10.4172/2155-6113.1000182

[19] Lichtenstein KA Ward DJ Moorman AC Delaney KM Young B Palella FJ. Clinical assessment of HIV-associated lipodystrophy in an ambulatory population. Aids. (2001) 15:1389–98. 10.1097/00002030-200107270-00008 11504960

[20] Scandlyn J. When AIDS became a chronic disease. Western J Med. (2000) 172:130. 10.1136/ewjm.172.2.130 10693378 PMC1070775

[21] Baril J-G Junod P LeBlanc R Dion H Therrien R Laplante F. HIV-associated lipodystrophy syndrome: A review of clinical aspects. Canad J Infect Dis Med Microbiol. (2005) 16:233–43. 10.1155/2005/303141 18159551 PMC2095035

[22] Sanches RS Mill J Machado AA Donadi EA Fernandes APM. Facial lipoatrophy: appearances are not deceiving. J Assoc Nurses AIDS Care. (2009) 20:169–75. 10.1016/j.jana.2009.01.002 19427594

[23] Lohman TG Roche AF Martorell R. Anthropometric Standardization Reference Manual. Chicago: Human Kinetics Books (1988).

[24] World Health Organization (WHO). Physical Status: the Use and Interpretation of Anthropometry. Geneva: WHO (1995).

[25] Ben-Noun L Sohar E Laor A. Neck circumference as a simple screening measure for identifying overweight and obese patients. Obesity Res. (2001) 9:470–7. 11500527 10.1038/oby.2001.61

[26] Naidu S Ponnampalvanar S Kamaruzzaman SB Kamarulzaman A. Prevalence of metabolic syndrome among people living with HIV in developing countries: a systematic review. AIDS Patient Care STDs. (2017) 31:1–13. 10.1089/apc.2016.0140 28051897

[27] Cuevas A Alvarez V Carrasco F. Epidemic of metabolic syndrome in Latin America. Curr Opin Endocrinol Diabetes Obesity. (2011) 18:134–8. 10.1097/MED.0b013e3283449167 21358406

[28] da Silva IC Sampaio E Almeida M Freire AN Sampaio LR Medeiros JMB. Perfil metabólico, antropométrico e lipodistrofia em pessoas vivendo com hiv/aids em uso de terapia antirretroviral. Nutr Clí*n Dietética Hospitalaria*. (2016) 36:38–44. 10.12873/363conceicaosilva

[29] Riddler SA Smit E Cole SR Li R Chmiel JS Dobs A. Impact of HIV infection and HAART on serum lipids in men. JAMA. (2003) 289:2978–82. 10.1001/jama.289.22.2978 12799406

[30] Rakotoambinina B Médioni J Rabian C Jubault V Jais J-P Viard J-P. Lipodystrophic syndromes and hyperlipidemia in a cohort of HIV-1-infected patients receiving triple combination antiretroviral therapy with a protease inhibitor. J Acquired Immune Deficiency Syndr. (1999) 27:443–9. 10.1097/00042560-200108150-00004 11511820

[31] Stevens J Katz EG Huxley RR. Associations between gender, age and waist circumference. Eur J Clin Nutr. (2010) 64:6–15. 10.1038/ejcn.2009.101 19738633 PMC5909719

[32] Berg G Mesch V Boero L Sayegh F Prada M Royer M. Lipid and lipoprotein profile in menopausal transition. Effects of hormones, age and fat distribution. Hormone Metab Res. (2004) 36:215–20. 10.1055/s-2004-814450 15114519

[33] Rönnemaa T Pulkki K Kaprio J. Serum soluble tumor necrosis factor-α receptor 2 is elevated in obesity but is not related to insulin sensitivity: a study in identical twins discordant for obesity. J Clin Endocrinol Metab. (2000) 85:2728–32. 10.1210/jcem.85.8.6720 10946872

[34] Hotamisligil GS Arner P Atkinson RL Spiegelman BM. Differential regulation of the p80 tumor necrosis factor receptor in human obesity and insulin resistance. Diabetes. (1997) 46:451–5. 10.2337/diab.46.3.451 9032102

[35] Christeff N Melchior JC De Truchis P Perronne C Gougeon ML. Increased serum interferon alpha in HIV-1 associated lipodystrophy syndrome. Eur J Clin Investig. (2002) 32:43–50. 10.1046/j.0014-2972.2001.00940.x 11851726

[36] Baron S Tyring SK Fleischmann WR Coppenhaver DH Niesel DW Klimpel GR. The interferons: mechanisms of action and clinical applications. JAMA. (1991) 266:1375–83. 10.1001/jama.1991.03470100067035 1715409

[37] Grunfeld C Kotler DP Shigenaga JK Doerrler W Tierney A Wang J. Circulating interferon-α levels and hypertriglyceridemia in the acquired immunodeficiency syndrome. Am J Med. (1991) 90:154–62. 10.1016/0002-9343(91)80154-E 1996584

[38] Grunfeld C Pang M Doerrler W Shigenaga J Jensen P Feingold K. Lipids, lipoproteins, triglyceride clearance, and cytokines in human immunodeficiency virus infection and the acquired immunodeficiency syndrome. J Clin Endocrinol Metab. (1992) 74:1045–52. 10.1210/jcem.74.5.1373735 1373735

[39] Bruun JM Lihn AS Verdich C Pedersen SB Toubro S Astrup A. Regulation of adiponectin by adipose tissue-derived cytokines: *in vivo* and *in vitro* investigations in humans. Am J Physiol Endocrinol Metab. (2003) 285:E527–33. 10.1152/ajpendo.00110.2003 12736161

[40] Engeli S Feldpausch M Gorzelniak K Hartwig F Heintze U Janke J. Association between adiponectin and mediators of inflammation in obese women. Diabetes. (2003) 52:942–7. 10.2337/diabetes.52.4.942 12663465

[41] Yudkin JS Stehouwer C Emeis J Coppack S. C-reactive protein in healthy subjects: associations with obesity, insulin resistance, and endothelial dysfunction: a potential role for cytokines originating from adipose tissue? Arteriosclerosis Thrombosis Vasc Biol. (1999) 19:972–8. 10.1161/01.ATV.19.4.972 10195925

[42] Biasucci LM Vitelli A Liuzzo G Altamura S Caligiuri G Monaco C. Elevated levels of interleukin-6 in unstable angina. Circulation. (1996) 94:874–7. 10.1161/01.CIR.94.5.874 8790019

[43] Berk BC Weintraub WS Alexander RW. Elevation of C-reactive protein in “active” coronary artery disease. Am J Cardiol. (1990) 65:168–72. 10.1016/0002-9149(90)90079-G 2296885

[44] Falasca K Ucciferri C Manzoli L Mancino P Pizzigallo E Conti P. Metabolic syndrome and cardiovascular risk in HIV-infected patients with lipodystrophy. Int J Immunopathol Pharmacol. (2007) 20:519–27. 10.1177/039463200702000310 17880765

[45] Bastard J-P Jardel C Bruckert E Blondy P Capeau J Laville M. Elevated levels of interleukin 6 are reduced in serum and subcutaneous adipose tissue of obese women after weight loss. J Clin Endocrinol Metab. (2000) 85:3338–42. 10.1210/jcem.85.9.6839 10999830

[46] Sankalé JL Tong Q Hadigan C Tan G Grinspoon S Kanki P. Regulation of adiponectin in adipocytes upon exposure to HIV-1. HIV Med. (2006) 7:268–74. 10.1111/j.1468-1293.2006.00372.x 16630040

[47] Ai M Otokozawa S Asztalos BF White CC Cupples LA Nakajima K. Adiponectin: an independent risk factor for coronary heart disease in men in the Framingham offspring Study. Atherosclerosis. (2011) 217:543–8. 10.1016/j.atherosclerosis.2011.05.035 21741045 PMC3662557

[48] Balmaceda JB Abd-Elmoniem KZ Liu CY Purdy JB Ouwerkerk R Matta JR. Brief report: adiponectin levels linked to subclinical myocardial fibrosis in HIV. JAIDS J Acquired Immune Deficiency Syndr. (2020) 85:316–9. 10.1097/QAI.0000000000002440 32639276

[49] Tong Q Sankalé J-L Hadigan CM Tan G Rosenberg ES Kanki PJ. Regulation of adiponectin in human immunodeficiency virus-infected patients: relationship to body composition and metabolic indices. J Clin Endocrinol Metab. (2003) 88:1559–64. 10.1210/jc.2002-021600 12679439

[50] Kershaw EE Flier JS. Adipose tissue as an endocrine organ. J Clin Endocrinol Metab. (2004) 89:2548–56. 10.1210/jc.2004-0395 15181022

[51] Nagy GS Tsiodras S Martin LD Avihingsanon A Gavrila A Hsu WC. Human immunodeficiency virus type 1-related lipoatrophy and lipohypertrophy are associated with serum concentrations of leptin. Clin Infect Dis. (2003) 36:795–802. 10.1086/367859 12627366

[52] Vigouroux C Maachi M Nguyên T-H Coussieu C Gharakhanian S Funahashi T. Serum adipocytokines are related to lipodystrophy and metabolic disorders in HIV-infected men under antiretroviral therapy. Aids. (2003) 17:1503–11. 10.1097/00002030-200307040-00011 12824788

[53] Group DCoAEoA-HDS. Combination antiretroviral therapy and the risk of myocardial infarction. N Engl J Med. (2003) 349:1993–2003. 10.1056/NEJMoa030218 14627784

[54] Friis-Møller N Weber R Reiss P Thiébaut R Kirk O Monforte AdA. Cardiovascular disease risk factors in HIV patients-association with antiretroviral therapy. Results from the DAD study. Aids. (2003) 17:1179–93. 10.1097/00002030-200305230-00010 12819520

